# Quercetin reduces oxidative stress and inhibits activation of c-Jun N-terminal kinase/activator protein-1 signaling in an experimental mouse model of abdominal aortic aneurysm

**DOI:** 10.3892/mmr.2013.1846

**Published:** 2013-12-06

**Authors:** LIAN WANG, XIAOFENG CHENG, HAO LI, FANG QIU, NAN YANG, BO WANG, HUCHEN LU, HAIWEI WU, YI SHEN, YANQING WANG, HUA JING

**Affiliations:** 1 Department of Thoracic Surgery, Second Affiliated Hospital, Zhejiang University School of Medicine, Hangzhou, Zhejiang 310009, P.R. China; 2 Department of Cardiothoracic Surgery, Jinling Hospital, Clinical Medicine School of Nanjing University, Nanjing, Jiangsu 210009, P.R. China; 3 D.A. Diagnostic Laboratory, Jinling Hospital, Clinical Medicine School of Nanjing University, Nanjing, Jiangsu 210009, P.R. China; 4 Department of Neurological Surgery, Jinling Hospital, Clinical Medicine School of Nanjing University, Nanjing, Jiangsu 210009, P.R. China; 5 Department of Cardiology, The 81st Hospital of PLA, Nanjing, Jiangsu 210002, P.R. China

**Keywords:** quercetin, abdominal aortic aneurysm, oxidative stress, nicotinamide adenine dinucleotide phosphate oxidase, c-Jun N-terminal kinase, activator protein-1

## Abstract

Oxidative stress is becoming increasingly linked to the pathogenesis of abdominal aortic aneurysms (AAAs). The antioxidant activity of flavonoids has attracted attention for their possible role in the prevention of cardiovascular diseases. The purpose of this study was to determine whether an antioxidant mechanism is involved in the aneurysm formation inhibitory effect afforded by quercetin. Male C57/BL6 mice received quercetin continuously from 2 weeks prior to and 6 weeks following the AAA induction with extraluminal CaCl_2_. Quercetin treatment decreased AAA incidence and inhibited the reactive oxygen species generation, nitrotyrosine formation and lipid peroxidation production in the aortic tissue during AAA development. In addition, quercetin-treated mice exhibited significantly lower expression of the p47phox subunit of nicotinamide adenine dinucleotide phosphate oxidase and inducible nitric oxide synthase, as well as coordinated downregulation of manganese-superoxide dismutase activities and glutathione peroxidase (GPx)-1 and GPx-3 expression. Quercetin also blunted the expression of c-Jun N-terminal kinase (JNK) and phospho-JNK and, in addition, diminished activation of the activator protein (AP)-1 transcription factor. Gelatin zymography showed that quercetin eliminated matrix metalloproteinase (MMP)-2 and MMP-9 activation during AAA formation. In conclusion, the inhibitory effects of quercetin on oxidative stress and MMP activation, through modulation of JNK/AP-1 signaling, may partly account for its benefit in CaCl_2_-induced AAA.

## Introduction

An abdominal aortic aneurysm (AAA) is a localized, permanent dilatation of the aorta that affects ~8% of males >65 years-old (1). At present, elective surgery is the major therapeutic option for AAA, however, this is not applicable to small aneurysms despite the reported growth rate of small aneurysms ranging between 1.5 and 3 mm per year, which leads to a higher risk of rupture (2). With increased knowledge of aneurysm pathophysiology, it is possible that aneurysm growth may be retarded with medical therapy.

The role of inflammation in the pathogenesis of AAA is well established. Infiltrating inflammatory cells enter the aorta, release cytokines and proteases, inducing apoptosis of vascular smooth muscle cells and ultimately, lead to destruction of the vascular wall (1). Moreover, the inflammatory microenvironment generates a large quantity of oxidant species, largely through upregulation of nicotinamide adenine dinucleotide phosphate (NADPH) oxidase in vascular cells (3). Furthermore, emerging evidence indicates that oxidative stress within the aortic wall is closely involved in the pathogenesis of AAA. Oxidative stress facilitates leukocyte recruitment into the vasculature by modulating adhesion molecules and chemotactic cytokines (4). In addition, reactive oxygen species (ROS) may alter the balance between destruction and regeneration of the aortic wall by enhancing matrix proteolysis through upregulation of matrix metalloproteinases (MMPs) (5). MMPs are the predominant extracellular proteinases that participate in the degradation process of structural proteins (1). Although human data remains limited, several studies indicate that antioxidant therapy may be effective in experimental AAA models (6–8).

Quercetin (3,5,7,3′4′-Pentahydroxy flavon), a typical member of the flavonoid family, is one of the most widely recognized dietary polyphenolic compounds. It is ubiquitously present in foods and is claimed to exert beneficial effects on vascular disease (9), which has been largely associated with its antioxidant and anti-inflammatory properties. Within the flavonoid family, quercetin is proven to be the most potent scavenger of free radicals (10). There is evidence that quercetin reduces low-density lipoprotein oxidation (11) and prevents the development of atherosclerotic lesions (12), in which oxidative stress is assumed to have a pivotal role. Although atherosclerosis and AAA are separate diseases, they have certain similar pathological characteristics, including inflammation and proteolysis (13). It is also reported that quercetin *in vitro* inhibits the production of O_2_^•−^ in the rat aorta and decreases protein expression of the NADPH oxidase subunit, p47phox (14,15). A previous study from our research group indicated that quercetin treatment inhibits inflammation and prevents CaCl_2_-induced aneurysmal dilation in a mouse AAA model (16). The present study was designed to test the hypothesis that an antioxidative mechanism is also involved in the protection afforded by quercetin.

## Materials and methods

### Pharmacological treatments

Quercetin was purchased from Sigma-Aldrich (Q4951; Shanghai, China). Drug solutions were prepared by suspending the compound in 0.5% carboxymethyl cellulose sodium. Animals were gavaged daily with 0.1 ml solution of quercetin (60 mg/kg) or vehicle alone, which began 2 weeks prior to AAA induction and continued for 8 weeks. The dose regimen for quercetin was based on previous studies demonstrating beneficial effects of the drug in mouse models of aortic atherosclerosis (12).

### Animal groups and the AAA model

A total of 60 male C57BL/6 wild-type mice (age, 6–7 weeks) were obtained from Vital River Laboratory Animal Technology (Beijing, China). All animals were treated and cared for in accordance with the Guide for the Care and Use of Laboratory Animals (National Institutes of Health, Washington DC, 1996) and the experimental protocols were approved by the Animal Care and Use Committee (Nanjing University, Nanjing, China). The mice were randomly assigned to one of four groups (n=15 in each group): Vehicle treatment plus sham operation control (VC), vehicle treatment plus AAA (VA), quercetin treatment plus AAA (QA) and quercetin treatment plus sham operation control (QC). AAA was induced in the infrarenal abdominal aorta (age, 8 weeks) by periaortic application of CaCl_2_, as previously described (16). NaCl (0.9%) was substituted for CaCl_2_ in sham operation animals. Six weeks later, the mice were laparotomied and the aortic diameters (ADs) were measured; the abdominal incision was carried upwards as a thoracoabdominal incision, the animals were then sacrificed by left-heart injection of potassium chloride and the aortic tissues were collected. An aneurysm was defined as an increase in the AD of >50% of the original AD.

### In vivo hemodynamic measurements

A computerized, non-invasive tail-cuff system with a four-channel mouse platform (BP-2000; Visitech Systems, Inc., Apex, NC, USA) was used to measure blood pressure and heart rate. To train mice, daily measurements were performed for five consecutive days prior to the actual recorded measurements. Hemodynamic parameters were measured one day pre- and 6 weeks post-AAA induction. The first 10 of 30 values recorded at each session were disregarded and the remaining 20 values were averaged and used for analysis, according to the manufacturer’s instructions.

### ROS analysis, lipid peroxidation determination and manganese-superoxide dismutase (Mn-SOD) activity assay

Dihydroethidium (DHE) oxidative fluorescence dye was used to evaluate *in situ* production of ROS (17). DHE stock solution was prepared by dissolving DHE (D7008; Sigma-Aldrich) in dimethylsulfoxide at a concentration of 5 mM. The stock solution was stored in the dark and diluted in phosphate-buffered saline (PBS) to a final concentration of 5 μM immediately prior to use. The abdominal aorta was harvested and the aortic segment (10 mm) was embedded in Tissue-Tek OCT compound (Sakura Finetech Japan, Tokyo, Japan) and snap-frozen. DHE working solution (200 μl) was topically applied to the aortic sections and the slides were subsequently incubated at 37°C in the dark for 30 min. Excess DHE was rinsed off twice with PBS and the images were immediately captured with a fluorescent microscope (BX51; Olympus, Tokyo, Japan) at excitation and emission wavelengths of 520 and 610 nm, respectively.

A portion of the snap-frozen aortic tissue (n=5 per group) was crushed in a prechilled mortar and resuspended in PBS at a concentration of 50 mg/ml. The homogenate was centrifuged at 10,000 × g for 10 min at 4°C to collect the supernatant. The lipid peroxidation product, malondialdehyde (MDA), was assessed using the thiobarbituric acid reactive substances (TBARS) assay kit (A003; Jiancheng Bioengineering, Shanghai, China). Briefly, 100 μl supernatant was added to 100 μl sodium dodecyl sulfate (SDS) lysis solution and mixed thoroughly. Following the addition of 250 μl thiobarbituric acid (TBA) reagent, samples were incubated at 95°C for 1 h and centrifuged at 1,500 × g at room temperature for 15 min. The absorbance of each supernatant was measured at 532 nm using a spectrophotometer (BioPhotometer; Eppendorf, Hamburg, Germany). Values of TBARS are expressed as nmol equivalents of MDA per mg protein. Mn-SOD activity was measured using an assay kit (A001-2; Jiancheng Bioengineering) according to the manufacturer’s instructions. Assay conditions were 65 μmol phosphate buffer (pH 7.8), 1 μmol hydrochloric hydroxylamine, 0.75 μmol xanthine and 2.3×10^−3^ IU xanthine dismutase. The supernatant (50 μl) was incubated in the system for 40 min at 37°C and terminated with 2 ml 3.3 g/l p-aminobenzene sulfonic acid and 10 g/l naphthylamine. For inhibition of CuZn-SOD activity, the assay was conducted in the presence of 10 mm KCN following preincubation for 30 min. The supernatant was transferred to a microplate (Eppendorf) for determination of the absorbance at 550 nm and 1 unit SOD was defined as the quantity of enzyme required to produce 50% dismutation of superoxide radical. Mn-SOD activity was calculated by subtraction of CuZn-SOD activity from total SOD activity. The standard curves were created as described in the manufacturer’s instructions. Images were assessed by Image J 1.44 software (National Institute of Health, Bethesda, MD, USA).

### Histological analysis

The infrarenal abdominal aorta (n=5 per group) was dissected and fixed in 10% neutral-buffered formalin. Specimens were dehydrated through graded ethanols, embedded in paraffin and sliced into 4–6-μm sections. Immunohistochemical staining with a rabbit polyclonal anti-nitrotyrosine antibody (1:500; 06–284; Millipore, Temecula, CA, USA) was used as an indicator of peroxynitrite formation (18). Briefly, the slides were incubated in 3% hydrogen peroxide for 5 min to quench endogenous peroxidase activity and were then incubated with primary antibody overnight at 4°C. Subsequently, slides were washed with PBS and incubated (15 min; 37°C) with peroxidase-conjugated goat anti-rabbit IgG (AP132P; Millipore). Finally, the slides were incubated with diaminobenzidine and counterstained with hematoxylin.

### Reverse transcription-polymerase chain reaction (RT-PCR)

RT-PCR was used to define the expression of glutathione peroxidase (GPx)-1, GPx-3, inducible nitric oxide synthase (iNOS) and p47phox NADPH oxidase mRNA. Total RNA was prepared with the TRIzol total RNA extraction kit (SK1321; Sangon Biotech, Shanghai, China). Primer sequences were as follows: Forward: 5′-ACC CCA AGT ACA TCA TTT GGT C-3′ and reverse: 5′-GCA GGG TTT CTA TGT CAG GTT C-3′ for GPx-1; forward: 5′-ATC TAC GAG TAT GGA GCC CTC A-3′ and reverse: 5′-GGC CCA AGT TCT TCT TGT AGT G-3′ for GPx-3; forward: 5′-CTT TGA CGC TCG GAA CTG TAG-3′ and reverse: 5′-AAC TCC AAG GTG GCA GCA T-3′ for iNOS; forward: 5′-CCC ATC ATC CTT CAG ACC TAT C-3′ and reverse: 5′-AAC CTC GCT TTG TCT TCA TCT G-3′ for p47 NADPH oxidase; and forward: 5′-AGG CCG GTG CTG AGT ATG TC-3′ and reverse: 5′-TGC CTG CTT CAC CAC CTT CT-3′ for GAPDH. Reverse transcription was performed with oligo-dT primers and the AMV First Strand cDNA Synthesis kit (SK2029; Sangon Biotech), according to the manufacturer’s instructions. The resultant cDNA was amplified by *Taq* DNA polymerase (SK2442; Sangon Biotech) in an Access RT-PCR System (Promega Corp., Madison, WI, USA). GAPDH mRNA was also amplified to serve as an internal control. The resultant PCR products were detected using an MSF-300G Scanner (Microtek Lab, Carson, CA, USA) and expressed as the ratio to GAPDH.

### Western blotting

Total protein was extracted from the supernatants of tissue homogenate with T-PER tissue protein extraction reagent (Pierce Biotechnology Inc., Rockford, IL, USA) and stored at −80°C. Equal quantities (30 μg) of total protein were separated on 10% polyacrylamide gels and transferred to nitrocellulose membranes using a semidry transfer cell (#164-5052; Bio-Rad, Hercules, CA, USA) at 10 V for 40 min. The membranes were blocked for 60 min with 5% nonfat milk in Tris-buffered saline with Tween-20 (TBST) and subsequently washed. Primary antibodies for p47phox NADPH oxidase (sc-14015), c-Jun N-terminal kinase (JNK; sc-571) and phosphorylated JNK (sc-6254) (all Santa Cruz Biotechnology, Santa Cruz, CA, USA) were added at a 1:500 dilution and incubated overnight at 4°C. Additionally, all blots were incubated with the anti-β-actin antibody (1:5,000; 4970; Cell Signaling Technology, Beverly, MA, USA) to confirm protein loading levels. Membranes were washed with TBST, incubated with horseradish peroxidase-conjugated species-appropriate secondary antibodies (Santa Cruz Biotechnology) for 1 h at room temperature and developed using an enhanced chemiluminescence kit (Pierce, Rockford, IL, USA). Quantification of images was performed by scanning densitometry with Image J 1.44.

### Electrophoretic mobility shift assay (EMSA)

Nuclear protein lysates were harvested using NE-PER nuclear and cytoplasmic extraction reagents (Pierce Biotechnology, Inc.) according to the manufacturer’s instructions. Activator protein (AP)-1 DNA-binding activities were analyzed using Gel Shift Assay systems (Promega Corporation, Madison, WI, USA), according to the instructions previously described (16). Briefly, the AP-1 consensus oligonucleotide probe (5′-CGC TTG ATG AGT CAG CCG GAA-3′) was end-labeled with [γ-^32^P]-ATP (Furui Biotech, Beijing, China). The extracted nuclear proteins (10 μg) were incubated for 20 min at 37°C with the ^32^P-labeled oligonucleotide (0.30 pmol) in a binding buffer. Reaction products were then separated in a 4% polyacrylamide gel, followed by autoradiography. The reactive bands were quantified as described in western blot analysis.

### Gelatin zymography

Protein extracts (10 μg) were mixed with SDS buffer and separated by electrophoresis on 10% SDS-polyacrylamide gels containing 1.0% gelatin. Following electrophoresis, the gels were renatured in renaturing buffer (LC2670; Invitrogen Life Technologies, Carlsbad, CA, USA) and incubated with developing buffer (LC2671; Invitrogen Life Technologies) for 30 min at room temperature. Subsequently, the gel was incubated in fresh developing buffer overnight at 37°C. The gel was stained with 0.5% Coomassie blue R-250 for 30 min and destained with destaining solution containing 10% acetic acid and 40% methanol. The relative molecular weight of each band was determined using protein standards (Pierce Biotechnology, Inc.). Areas of protease activity appeared as unstained bands against a blue background. Images were assessed by Image J 1.44.

### Statistical Analysis

All values are expressed as the mean ± standard deviation. Statistical analyses were performed with SPSS for Windows version 17.0 (SPSS, Inc., Chicago, IL, USA). Within-group comparisons of hemodynamic parameters at various intervals were performed using paired Student’s t tests. Between-group comparisons were performed using the Fisher’s exact test or analysis of variance. P<0.05 was considered to indicate a statistically significant difference.

## Results

### AAA incidence

No significant difference was found in AD at the time of surgery among the groups (data not shown). Six weeks later, the VA mice showed a marked increase in AD following CaCl_2_ treatment with 10/15 (66.7%) developing aneurysms. Only 3/15 (20%) of the aortas became aneurysmal in QA mice; this difference in aneurysm incidence was considered to be highly significant (P<0.05). No aneurysm formation was observed in the VC or QC mice. Quercetin treatment had no effect on mean arterial pressure or heart rate when measured pre- and post-surgery (Table I).

### ROS generation, nitrotyrosine formation and lipid peroxidation production

**Figure 1:**
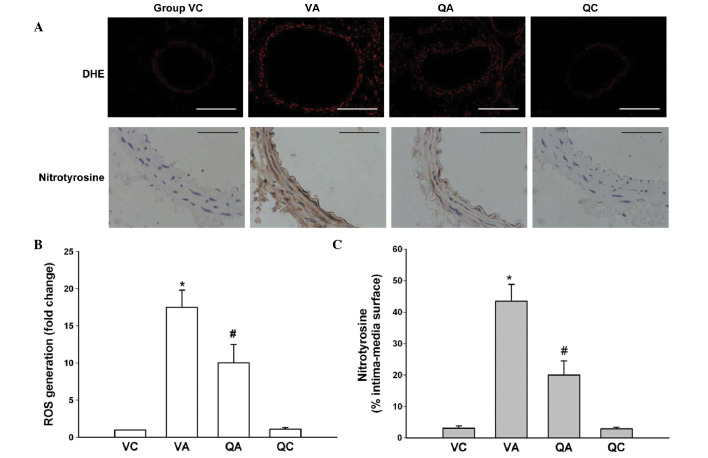
Effect of quercetin on ROS generation and nitrotyrosine formation in the aortic wall. (A) DHE fluorescence imaging and immunohistochemical staining for nitrotyrosine in the mouse aortic wall (details in Materials and methods). Scale bars are 500 and 50 μm, respectively. (B) Densitometric analysis of DHE fluorescence relative to VC mice. (C) Positively stained areas in the aortic section of each group. Values are presented as the mean ± standard deviation with n=5 per group. ^*^P<0.05, vs. VC; ^#^P<0.05, vs. VA. ROS, reactive oxygen species; DHE, dihydroethidium; AAA, abdominal aortic aneurysm; VC, vehicle treatment plus sham operation control; VA, vehicle treatment plus AAA; QA, quercetin treatment plus AAA; QC, quercetin treatment plus sham operation control.

To evaluate the effect of quercetin on ROS generation, aortic sections were exposed to DHE, which is transformed to the highly fluorescent molecule, oxyethidium, in the presence of superoxide (17). As shown in Fig. 1A, ROS production was extremely low in aortas from VC and QC mice. At 6 weeks post-AAA induction in VA mice, oxyethidium fluorescence was higher, being significantly enhanced throughout the vascular wall (Fig. 1A and B). However, it was attenuated in QA mice, indicating decreased ROS production due to quercetin treatment.

As increased production of ROS may lead to further peroxynitrite accumulation, which induces protein damage by formation of nitrotyrosine (18), immunohistochemistry was performed with a polyclonal antibody against nitrotyrosine in aortic cross sections. Staining appeared only weakly in the aorta of VC and QC animals, however, VA mice revealed marked brown nitrotyrosine staining in the aortic wall. By contrast, a decreased immunoreactivity was observed in QA mice (Fig. 1A and C). Similar observations were noted for lipid peroxidation production (MDA) levels (TBARS) in aortic tissues. The TBARS concentration in QA mice was found to be significantly lower than that in the VA mice (Table II).

### Endogenous vascular antioxidant defense systems

**Figure 2:**
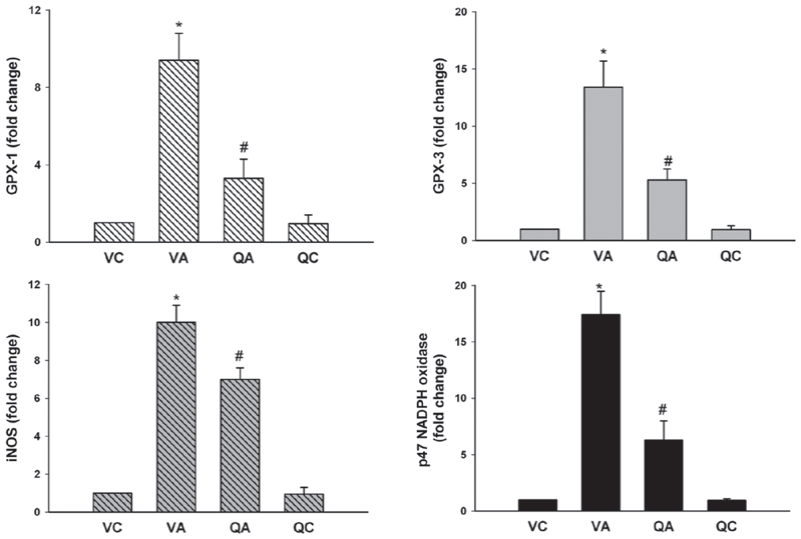
mRNA expression of GPx-1, GPx-3, iNOS and p47 NADPH oxidase. Data are expressed as fold changes compared with values of VC animals (n=5 in each group). ^*^P<0.05, vs. VC; ^#^P<0.05, vs. VA. AAA, abdominal aortic aneurysm; iNOS, inducible nitric oxide synthase; Gx, glutathione peroxidase; NADPH, nicotinamide adenine dinucleotide phosphate; VC, vehicle treatment plus sham operation control; VA, vehicle treatment plus AAA; QA, quercetin treatment plus AAA; QC, quercetin treatment plus sham operation control.

Increased levels of Mn-SOD activity were observed at AAA regions of the VA mice, while quercetin significantly decreased its activity in QA mice (Table II). In addition, quercetin caused a relative decrease in mRNA expression of GPx-1 and GPx-3, which are involved in antioxidative status (Fig. 2).

### Expression of iNOS and p47phox NADPH oxidase

**Figure 3:**
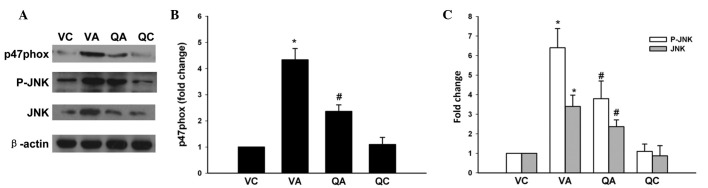
Expression of p47phox NADPH oxidase, phosphorylated JNK and total JNK (A). Data are presented as the mean ± standard deviation with n=5 per group and expressed as fold changes compared with values in the VC animals (B). ^*^P<0.05, vs. VC; ^#^P<0.05, vs. VA. AAA, abdominal aortic aneurysm; JNK, c-Jun N-terminal kinase; NADPH, nicotinamide adenine dinucleotide phosphate; VC, vehicle treatment plus sham operation control; VA, vehicle treatment plus AAA; QA, quercetin treatment plus AAA; QC, quercetin treatment plus sham operation control.

Expression of iNOS and the NADPH oxidase subunit, p47phox, was also examined. Compared with the VA group, QA mice showed relative decreases in iNOS and p47phox mRNA levels (Fig. 2) and this result was confirmed by western blot analysis of p47phox (Fig. 3A and B).

### JNK/AP-1 signaling pathway and enzymatic activities of MMPs

**Figure 4:**
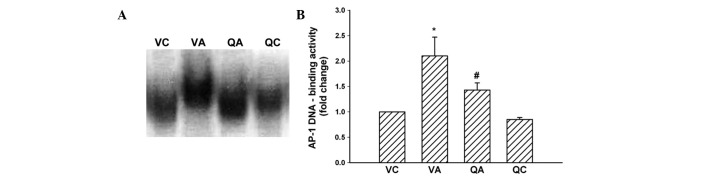
EMSA for AP-1 binding sites. (A) Representative results of EMSA for AP-1 binding sites 6 weeks following NaCl/CaCl_2_ incubation. (B) Quantification of the hybridization shown in panel (A) by densitometric analysis. Signals from animals in the VC group were arbitrarily provided as 100%; those from other animals were expressed as the percentage of values in the VC animals. n=5 per group. ^*^P<0.05, vs. VC; ^#^P<0.05, vs. VA. EMSA, electrophoretic mobility shift assay; AP-1, activator protein; AAA, abdominal aortic aneurysm; VC, vehicle treatment plus sham operation control; VA, vehicle treatment plus AAA; QA, quercetin treatment plus AAA; QC, quercetin treatment plus sham operation control.

Since quercetin reduced oxidative stress, the specific contribution of quercetin to the regulation of AP-1 activation in experimental AAA was examined. Levels of AP-1 DNA binding activity in QA mice, determined by EMSA, were significantly inhibited by quercetin when compared with controls in the VA group (Fig. 4A and B). Western blotting showed that phosphorylated-JNK was significantly upregulated in VA mice and downregulated following treatment with quercetin. In addition, QA animals had significantly less total JNK than VA controls (Fig. 3A and B).

**Figure 5:**
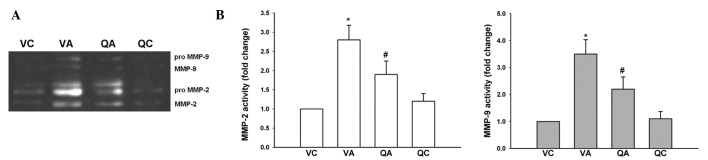
Gelatin zymography analysis of MMP-2 and -9 activities. (A) Representative gelatin zymography at 6 weeks following NaCl/CaCl_2_ incubation. (B) Calculated relative density (percentage of VC) of gelatin zymography showed differences in MMP activities between groups (n=5 per group). ^*^P<0.05, vs. VC; ^#^P<0.05, vs. VA. MMP, matrix metalloproteinase; AAA, abdominal aortic aneurysm; VC, vehicle treatment plus sham operation control; VA, vehicle treatment plus AAA; QA, quercetin treatment plus AAA; QC, quercetin treatment plus sham operation control.

Gelatin zymography revealed that MMP-2 and -9 activities were elevated in VA mice, however, quercetin treatment resulted in a marked decrease in MMP-2 and -9 activities (Fig. 5).

## Discussion

The CaCl_2_-induced AAA model has been widely employed to gain further understanding of the mechanisms involved in aneurysm development, in order to identify potential novel medical treatments (19). As the results of this study showed, the development of CaCl_2_-induced AAA in mice was accompanied by elevated aortic ROS levels, increased nitrotyrosine formation and lipid peroxidation products, indicating an enhancement in overall oxidative stress. Previous studies have demonstrated that markers of oxidative damage are present in human (20) and animal (21) aneurysmal lesions. However, these oxidative stress markers were significantly inhibited by supplementation of quercetin, a dietary antioxidant with a polyphenolic structure. The antioxidant activity of polyphenols has attracted much attention in relation to their possible role in the prevention of chronic diseases (22). In particular, it was previously reported that resveratrol, another polyphenolic compound, counteracts systemic (23) and local (24) oxidative stress and limits experimental AAA progression. Moreover, a variety of medications and interventions have been proven to successfully suppress experimental aneurysm formation through a ROS-based mechanism (6–8). Thus, it was hypothesized that the aneurysm-inhibitory effect of quercetin in the present study may, in part, associate with its lower oxidation-reduction potential.

Oxidative stress is the result of a redox imbalance between the generation of ROS and the secondary response from the endogenous antioxidant network. Results from the present study indicate a local upregulation of the endogenous antioxidant system, including Mn-SOD and GPxs during CaCl_2_-induced AAA formation. Mn-SOD and GPxs are key scavengers of ROS, for example, H_2_O_2_ and lipid hydroperoxides (25,26). Therefore, increases in Mn-SOD and GPxs may be a compensatory response for an increase in ROS in the mouse aorta following exposure to CaCl_2_. Quercetin, by restraining ROS levels, prevents the elevation of those antioxidant enzymes, coinciding with other study results (27–29), which have reported the protective effect of quercetin on organ injury.

The enhanced expression of NADPH oxidase, an enzyme that catalyzes the production of O_2_^•−^ from oxygen and NADPH, is a major pathway of ROS formation in the vascular wall (3). Inhibition of ROS production by oral administration of apocynin, a specific inhibitor of NADPH oxidases, attenuates AAA formation in a murine model (8). It was also reported that quercetin prevented the increase in aortic O_2_^•−^ production through downregulation of p47phox expression *in vivo* and *in vitro* (14,15). Furthermore, Thomas *et al* (30) have shown that p47phox deficiency reduced oxidative stress and markedly attenuated AAA formation. The present study found that quercetin treatment significantly eliminated gene and protein expression of p47phox NADPH oxidase and these data, together, demonstrate that quercetin is able to reduce ROS formation via modulation of the p47phox subunit during AAA development.

It is noteworthy that, in a previous study, iNOS deficient mice were partly resistant to aneurysm induction by CaCl_2_ (8). Nitric oxide synthase (NOS) is also a source of ROS and increased cellular expression of iNOS is specifically associated with large quantities of nitric oxide produced during chronic inflammation (31). The present study found that expression of iNOS in the aortic wall was inhibited following quercetin treatment. It has been reported that under inflammation-mimicking conditions, quercetin may inhibits iNOS expression in cultured monocytes (32,33). This result indicates another mechanism through which quercetin may impact ROS generation.

More importantly, oxidative stress has been reported to activate MMPs (34,35), a family of enzymes with the capacity to cleave several components of the extracellular matrix, including elastin and collagen. It is generally hypothesized that MMPs are putative therapeutic targets in the prevention of AAA (1). Our study group has previously reported that treatment of mice with quercetin prevents aortic wall destruction in the CaCl_2_-induced AAA model, which is associated with a reduction in the expression of MMP-2 and -9 (16). In the current study, similar results were observed when determining the enzymatic activities of MMPs *in vitro* by gelatin zymography. MMPs are primarily regulated at the gene transcriptional level by various factors, including cytokines, growth factors, ROS and reactive nitrogen species (RNS). AP-1, a major downstream target of JNK, is an essential transcription factor for MMP expression (36–38). The present study shows that quercetin treatment significantly inhibited AP-1 activation, accompanied by decreased phosphorylation of JNK in AAA tissues. JNK, also known as stress-activated protein kinase, is hypothesized to be involved in a number of cellular stress responses. It is well established that ROS produced from NADPH oxidase and RNS are potent inducers of JNK (39). Moreover, the existing evidence indicates that JNK has an important role in AAA. Yoshimura *et al* (40) have demonstrated that pharmacological inhibition of JNK reduces MMP levels and prevents the development of AAA. Furthermore, JNK inhibition caused regression of established aneurysm in CaCl_2_- and angiotensin II-infusion-induced AAA models. Activation of JNK leads to modulation of other kinases, their nuclear translocation and subsequent phosphorylation of a number of transcription factors, including AP-1 (41). Thus, data from the present study indicated that quercetin reduces oxidative stress and blocks aneurysm formation, which may occur via the mediation of the JNK/AP-1 pathway and MMP modulation.

In conclusion, the present study demonstrated that an antioxidative mechanism is involved in the preventive action of quercetin on CaCl_2_-induced AAA. This is notable as AAA is a chronic and serious condition for which no medical treatment currently exists. In addition, the compound has been observed to be effective in reducing the risk factors of cardiovascular disease that often occur simultaneously with AAA (1,9). Although it is unclear whether these experimental observations extend to aneurysmal degeneration as it occurs in humans, it is likely to be a point of interest to explore in future investigations.

## Figures and Tables

**Table I:** Heart rate and mean arterial pressure prior to and following NaCl/CaCl_2_ treatment.

	2 weeks prior to surgery		Surgery		6 weeks post-surgery	
			
Group (n=15)	HR, bpm	MAP, mmHg	HR, bpm	MAP, mmHg	HR, bpm	MAP, mmHg
VC	630±30	83±8	626±34	85±6	633±27	85±6
VA	628±28	85±7	632±29	82±5	631±35	86±9
QA	631±33	84±6	629±33	84±8	627±34	83±5
QC	634±31	84±9	633±34	85±5	630±32	85±8

Heart rate and mean arterial pressure were recorded using the tail-cuff system. Measurements are expressed as the mean ± standard deviation. AAA, abdominal aortic aneurysm; VC, vehicle treatment plus sham operation control; VA, vehicle treatment plus AAA; QA, quercetin treatment plus AAA; QC, quercetin treatment plus sham operation control.

**Table II:** Effect of quercetin on MDA levels and Mn-SOD activities following NaCl/CaCl_2_ incubation.

Group	TBARS (nm MDA/mg protein)	Mn-SOD activities (U/mg protein)
VC	0.075±0.027	2.8±1.5
VA	0.342±0.035^a^	28.2±5.4^a^
QA	0.231±0.029^b^	15.5±4.9^b^
QC	0.069±0.015	2.5±1.8

Values are presented as the mean ± standard deviation with n=5 per group.

a P<0.05 vs. VC;

b P<0.05 vs. VA.

AAA, abdominal aortic aneurysm; MDA, malondialdehyde; Mn-SOD, manganese-superoxide dismutase; TBARS, thiobarbituric acid reactive substances (assay kit); VC, vehicle treatment plus sham operation control; VA, vehicle treatment plus AAA; QA, quercetin treatment plus AAA; QC, quercetin treatment plus sham operation control.
